# Resveratrol and Vitamin D: Eclectic Molecules Promoting Mitochondrial Health in Sarcopenia

**DOI:** 10.3390/ijms25147503

**Published:** 2024-07-09

**Authors:** Cristina Russo, Maria Stella Valle, Floriana D’Angeli, Sofia Surdo, Lucia Malaguarnera

**Affiliations:** 1 Section of Pathology, Department of Biomedical and Biotechnological Sciences, School of Medicine, University of Catania, 95123 Catania, Italy; cristina.russo@unict.it; 2 Section of Physiology, Department of Biomedical and Biotechnological Sciences, University of Catania, 95123 Catania, Italy; m.valle@unict.it; 3 Department of Human Sciences and Quality of Life Promotion, San Raffaele Roma Open University, 00166 Rome, Italy; floriana.dangeli@uniroma5.it; 4 Italian Center for the Study of Osteopathy (CSDOI), 95124 Catania, Italy; s.surdo98@yahoo.com

**Keywords:** resveratrol, vitamin D, oxidative stress, mitochondria, muscle homeostasis, muscle dysfunction

## Abstract

Sarcopenia refers to the progressive loss and atrophy of skeletal muscle function, often associated with aging or secondary to conditions involving systemic inflammation, oxidative stress, and mitochondrial dysfunction. Recent evidence indicates that skeletal muscle function is not only influenced by physical, environmental, and genetic factors but is also significantly impacted by nutritional deficiencies. Natural compounds with antioxidant properties, such as resveratrol and vitamin D, have shown promise in preventing mitochondrial dysfunction in skeletal muscle cells. These antioxidants can slow down muscle atrophy by regulating mitochondrial functions and neuromuscular junctions. This review provides an overview of the molecular mechanisms leading to skeletal muscle atrophy and summarizes recent advances in using resveratrol and vitamin D supplementation for its prevention and treatment. Understanding these molecular mechanisms and implementing combined interventions can optimize treatment outcomes, ensure muscle function recovery, and improve the quality of life for patients.

## 1. Introduction

Sarcopenia is characterized by progressive and generalized skeletal muscle weakness, leading to rapid muscle failure and functional deterioration [1]. This condition significantly reduces the quality of life for millions worldwide and is associated with increased mortality and morbidity [2]. Sarcopenia can be classified into primary and secondary types. Primary sarcopenia, associated with aging, has become a major health issue among the elderly [3], resulting in disability, increased risk of falls, and mortality [4]. Secondary sarcopenia, on the other hand, arises from the co-morbidities of various inflammatory and chronic degenerative diseases. These include type 2 diabetes, metabolic disorders, cardiovascular diseases, chronic obstructive pulmonary disease, kidney and liver diseases, microvascular injury, reduced satellite cell numbers, neurodegenerative disorders, and cancer [5]. Additional latent causes of secondary sarcopenia include excess body fat, malnutrition, prolonged hospital stays, and inactivity [6].

Any pathological condition characterized by systemic inflammation and oxidative stress can impair muscle function through enzymatic changes and mitochondrial abnormalities [7]. Oxidative stress is particularly relevant in skeletal muscle damage. At physiological levels, free radicals, such as reactive oxygen species (ROS) and reactive nitrogen species (RNS), are essential for regulating cellular processes to maintain homeostasis. These processes include cell signaling, division, proliferation, regeneration, and apoptosis. For instance, after an injury, myocytes require optimal ROS levels for signal transduction. However, excessive ROS production, coupled with inadequate antioxidant defenses, impairs muscle tissue health [5]. The overproduction of free radicals depends on various causes, including changes in cellular metabolism, pollution, and wrong lifestyle habits such as malnutrition and smoking. The balance between free radicals from metabolism or environmental sources and the failure of antioxidant defense systems—such as superoxide dismutase (SOD), catalase, and glutathione peroxidase (GPx)—is crucial in the development and progression of sarcopenia [7]. Free radicals generate low-grade inflammation, accompanied by the production of mediators that stimulate inflammatory cells and generate a vicious circle with a further progression of muscle atrophy and loss. Moreover, deleterious oxidative variation of macromolecules generates an alteration of signal transduction pathways that control mitochondrial homeostasis, energetic metabolism, antioxidant gene expression, and redox balance, leading to myocyte dysfunctions. Many aspects of mitochondrial dysfunction and bio-energetic failure are accountable for the development of different types of sarcopenia [8]. Mitochondrial biogenesis regulation depends on physiological conditions and energetic changes. Cells and tissues respond to increased energy demands by accelerating the formation of new mitochondria. Thus, it is not surprising that the availability of nutrients, environmental pollutants, hormonal fluctuations, exercise, hypoxia, stress, and aging influence the process of mitochondriogenesis [5]. Skeletal muscle function is strongly influenced by nutritional and genetic factors. Natural compounds with antioxidant properties may help counteract oxidative stress by neutralizing excess free radicals and preventing the early chain reactions that promote disease onset [9]. In this context, in recent decades, several experimental investigations have identified resveratrol and vitamin D as promising compounds that preserve mitochondrial function in skeletal muscle cells. This review provides an overview of the molecular mechanisms through which resveratrol and vitamin D promote mitochondrial health in sarcopenia. It highlights their importance as adjunct therapeutic supplements in managing muscle weakness in elderly and frail individuals, aiming to delay the progression of sarcopenia.

## 2. Molecular Mechanisms of Myogenesis in Adulthood

The adult skeletal muscle possesses an intrinsic capability for regeneration. Myogenesis, which allows muscle regeneration in adulthood, is a multifaceted and well-coordinated event. It regulates the homeostasis of skeletal muscle tissue and ensures the restoration of its mass and function. It is regulated by a set of protein kinases activated by pro-myogenic stimuli and a reversible phosphorylation process that produces signals. Myogenesis occurs through the regulation of gene expression by different transcription factors, including the MyoD family of basic helix–loop–helix myogenic regulatory factors (MRFs), MyoD, Myf5, MRF4, and myogenin, which are the master regulators of myogenesis [10]. After muscle injury, or degeneration, muscle-resident stem cells, namely satellite cells (SCs), are activated and proliferate. Both SC and myogenic precursor cells are able to form myoblasts; they proliferate by expressing myogenic regulatory factors, before leaving the cell cycle, and undergoing morphological changes. SCs reside in a defined anatomical hypoxic niche and help to maintain the architecture and functions of skeletal muscle [11]. In hypoxic niches, quiescent SCs express to a lesser extent PAX3 and, to a greater extent, PAX7, which has been defined as a matrix regulator of SC function since it regulates the expression of genes involved in their survival, proliferation, and differentiation [12].The balance between PAX7 and MyoD determines the fate of SCs. PAX7 overexpression prevents differentiation by inhibiting MyoD and myogenin, restraining myoblast fusion with impaired myofibers to achieve regeneration [13]. Once SCs start to differentiate into myoblasts, other particular differentiation markers are upregulated, such as myogenin, slow-twitch skeletal muscle Ca^2+^-ATPase, various isoforms of myosin heavy chain (MHC), and insulin-like growth factors (IGFs), which play a decisive role in SC differentiation to form contractile myofibers for muscle regeneration [14]. Myoblasts are highly proliferative cells characterized by the expression of MRF, in particular MyoD, which is crucial for adult muscle regeneration [15]. Myogenin is a direct target of MyoD and facilitates complete differentiation of myogenic progenitor cells [15]. Almost all stages of muscle regeneration are strongly regulated by acute inflammation and immune cells that produce cytokines that support the activities of SCs, determining the effectiveness of regeneration [16]. The involvement of these factors in the differentiation phase suggests that skeletal muscle regeneration is controlled by a complex mechanism in which each element plays a crucial role. Along with these factors, macrophages play a central role in regulating skeletal muscle regeneration by giving rise to M1 pro-inflammatory and M2 macrophages, which are tissue-healing [17]. M1 macrophages are the predominant type of macrophages in the early phase of muscle regeneration. They remove muscle debris resulting from trauma and secrete cytokines, such as interleukin 6 (IL-6) and tumor necrosis factor α (TNF-α) [18]. TNF-α recruits SCs to the damaged muscle site, and by activating the transcription factor nuclear factor-kappa B (NF-κB) and the p38 signaling pathway, it promotes SC proliferation and differentiation [19]. Nevertheless, a strong activation of NF-κB in myoblasts has been shown to inhibit MyoD synthesis and upregulate PAX7 [20]. It has been observed that PAX7 upregulation exacerbates muscle mass decline and increases muscle wasting in cancer cachexia, while inhibition of NF-κB signaling rescues cancer-related atrophy [21]. SCs differentiation in newly formed myofibers is sustained by the switch to an anti-inflammatory microenvironment supported by M2 macrophages, which produce anti-inflammatory cytokines such as IL-4, IL-10, and IL-13, reverse the local inflammatory response at the injury site [22]. This conversion is an essential component of muscle regeneration in vivo after acute or chronic muscle injury. The M1 to M2 switch is facilitated by the CREB-C/EBPbeta signal and by IL-10, secreted by regulatory T cells (Tregs) [23]. Genetic depletion of C/EBPβ leads to increased apoptosis and further impairment of regeneration. In vitro studies suggest that TNF-α can induce apoptosis and thus modulate the pool of functional SCs [24]. M2 macrophage conversion promotes myotube formation and muscle regeneration [25]. The absence of M2 macrophages blocks muscle differentiation and regeneration, generating a deferral in muscle growth. In addition to macrophages, T cells are the major cell population recruited to the lesion in the second flow of immune cell infiltration. Cytokines such as TNF-α, IL-1α, IL-13, and IFN-γ secreted by T cells facilitate SC expansion both in vivo and in vitro [26]. Following an injury, the concentration of oxygen in muscle tissue and niches changes, and thus the expression of inducible hypoxia factors (HF) [27].

In fact, the release of cytokines, neurotrophic factors, growth factors, or oxygen tension facilitating the hypoxia-inducible gene program such as Hif1α, Hif2α, and NO cooperatively controls the status of the satellite cell pool.

HIF1α improves skeletal muscle regeneration and leads to the formation of larger fibers [28]. On the other hand, HIF2α levels decrease sharply after an injury and gradually return to their basic level after muscle regeneration [27]. The expression of Hif1α triggers Notch downstream genes to preserve the stem/progenitor cell state [29].

## 3. Resveratrol and Skeletal Muscle Function

Resveratrol (3,5,4′-trihydroxy-trans-stilbene) is a natural polyphenol found in various plant species, predominantly in red wine, red grapes, rhubarb, mulberries, and peanuts [30]. It is known for its significant role in preventing or slowing the progression of various diseases due to its antioxidant, anti-inflammatory, anticancer, antimicrobial, anti-neurodegenerative, and estrogenic properties [31].

**Figure 1:**
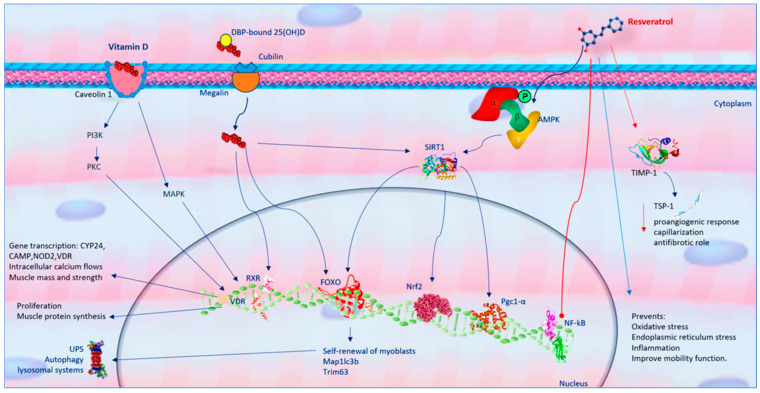
Similarity and difference of resveratrol and vitamin D on skeletal muscle function. Red arrows show inhibition processes, while blue arrows show activation processes. Abbreviations: adenosine monophosphate (AMP); adenosine monophosphate protein kinase (AMPK); forkhead box O1 (FoxO1); nuclear factor kappa-light-chain-enhancer of activated B cells (NF-κB); metallopeptidase inhibitor-1 (TIMP-1); microtubule-associated proteins 1A/1B light chain 3B (MAP1LC3B); nuclear factor related erythroide factor 2 (Nrf2); phosphoinositide 3-kinase (PI3K); protein kinase C (PKC); sirtuin 1 (SIRT-1); tripartite motif containing 63 (TRIM63); thrombospondin-1 (TSP-1); ubiquitin–proteasome system (UPS); vitamin D receptor (VDR).

Resveratrol effects different muscle structures, such as skeletal muscle fibers, neuromuscular junction (NMJ), connective sheaths, tendons, and vessels, potentially preventing muscle wasting in various conditions. Research has demonstrated that resveratrol exerts a critical role in contractile protein accumulation and muscle strength, improves muscle fiber cross-sectional area, and contributes to satellite cell proliferation in aged muscle tissue [32,33]. Moreover, resveratrol could directly stimulate protein synthesis, inhibit lysosomal and ubiquitin–proteasome system (UPS) systems, and inhibit protein degradation in innervated and denervated muscles by activating the PKA/CREB pathway [34]. Acting on myofibers by modulating metabolism, catabolism, and oxidative stress, resveratrol exerts beneficial effects on skeletal muscles both in vitro and in vivo. However, the data are conflicting due to differences in dosage and treatment duration. Some studies report that resveratrol inhibits protein degradation and attenuates skeletal muscle atrophy, reducing age-dependent fiber area loss [35,36]. In elderly animals with sarcopenia, resveratrol supplementation has been shown to improve muscle mass and function [37]. It also inhibits the phosphatidylinositol 3-kinase (PI3K)/Akt pathway, which regulates cell differentiation, growth, and proliferation [38]. Despite its protective effects against aging-induced oxidative stress, some studies suggest that resveratrol does not mitigate sarcopenia [39]. The beneficial effects of resveratrol in skeletal muscle are largely due to its interaction with signaling pathways that modulate cellular metabolism, inhibit protein catabolism, and protect against cellular stress [40]. Resveratrol exerts its metabolic effects mainly through the activation of adenosine monophosphate (AMP)-activated protein kinase (AMPK), the consequent modulation of a signaling cascade involving SIRT-1, forkhead box O1 (FoxO1), a nuclear factor related erythroid factor (Nrf) 2, and other effectors [30]. In addition, resveratrol controls cyclooxygenases [41], directly or indirectly eliminates ROS and RNS [42], and acts as phytoestrogens [43]. In its antiproteolytic action, resveratrol may control FoxO through different post-translational changes such as phosphorylation, acetylation, and ubiquitination [44]. In skeletal muscle, FoxO proteins induce the transcription of numerous atrogenes, such as *Map1lc3b*, *Ctsl*, *Fbxo32*, and *Trim63*, improving the activity of UPS and autophagy–lysosomal systems [44]. FoxO, in turn, may be regulated by several intracellular pathways, including insulin/insulin-like growth factor-1 (IGF-1)/phosphatidylinositol 3-kinase (PI3K)/Akt signaling [45]. Resveratrol can inactivate Akt/FoxO4 signaling, reducing Akt-induced phosphorylation of FoxO in muscle [44]. Resveratrol prevents protein degradation by interfering with nuclear factor kappa B (NF-κB) activation and nuclear translocation [30] and constraining the signaling pathway that induces muscle mass waste [46]. Resveratrol inhibits NF-κB and its nuclear translocation, reducing cytokines and inflammatory markers [47], inhibiting protein degradation [48], and signaling pathways that contribute to muscle mass loss [46]. Resveratrol prevents the training-induced expression of the metallopeptidase inhibitor-1 (TIMP-1) and reduces the levels of thrombospondin-1 (TSP-1), suggesting that it inhibits the proangiogenic response and capillarization [49]. Since TIMP-1 inhibits the function of both metalloproteinase and TSP-1 in promoting fibrosis [50], it is conceivable that in skeletal muscle, resveratrol plays an antifibrotic role. Additionally, resveratrol prevents oxidative stress, endoplasmic reticulum stress, and inflammation, decreasing nerve cell senescence, exerting neuroprotective properties, and improving mobility function [51] (Figure 1).

## 4. Vitamin D and Skeletal Muscle Function

Vitamin D, or calciferol, exists in two active forms: D3 (cholecalciferol) and D2 (ergocalciferol). Vitamin D3, the primary endogenous source, is synthesized in the skin from 7-dehydrocholesterol (7DHC) through ultraviolet B (UVB) radiation exposure. Vitamin D can also be obtained from dietary sources, with mushrooms containing vitamin D2 and foods like bluefish, eggs, and liver providing vitamin D3 [52]. Enriching daily foods with vitamin D2 and D3 supplements provides an additional dietary pathway for vitamin D assimilation. Both vitamin D3 and D2 follow the same metabolic pathway to synthesize the biologically active form. The first metabolic phase occurs in the liver, where D2/D3 is converted into 25-hydroxyvitamin D (25(OH)D; calcidiol) via 25-hydroxylase (CYP2R1) [53]. Calcidiol, the main circulating form of vitamin D, is commonly used to clinically assess plasma vitamin D levels. Most circulating 25(OH)D is bound to a protein carrier, the vitamin D binding protein (DBP), belonging to the albumin family [54]. DBP-bound 25(OH)D is transported to the kidneys, where it is hydroxylated by 1α-hydroxylase (CYP27B1) to form, 1α,25-dihydroxyvitamin D (1,25(OH)2D; calcitriol), the biologically active metabolite. Mainly, vitamin D is a regulator of Ca2+ and preserves skeletal health. It also has significant immunoregulatory effects, influencing inflammatory responses, muscle damage, and aerobic capacity [7]. The importance of vitamin D for muscle activity was first evidenced by the severe muscle damage observed in children with rickets, known as “rachitic myopathy” [55]. A key role in the pathophysiological mechanisms of muscle damage is carried out by variations in the concentration of phosphoric ester in muscle tissue [56]. In addition, the effects of hyperparathyroidism secondary to hypovitaminosis D on skeletal muscle are important. Hyperparathyroidism causes atrophy of muscle tissue due to a preferential loss of type II fibers, which differs from pathological alterations of primary myopathy, in which degeneration occurs, until the necrosis of muscle fibers, accompanied by the proliferation of endomisial connective tissue [57]. In its active form, calcitriol affects muscle function by regulating intracellular calcium levels through voltage-dependent SOC/TRPC3 channels, influencing the excitation–contraction coupling of skeletal muscle fibers. In skeletal muscle, DBP-bound 25(OH)D enters target cells through the megalin-cubilin transmembrane complex. Within the cell, the D-DBP complex binds to cytoplasmic actin. The muscle actions of vitamin D are due to the expression of vitamin D system-related proteins such as vitamin D receptors (VDRs), CYP27B1, and CYP24A1 in skeletal muscle tissue. These proteins are expressed in skeletal muscle stem cells (satellite cells) after injury and during muscle regeneration, indicating vitamin D’s direct role in muscle repair and maintenance [58]. VDR in musculoskeletal tissue facilitates muscle protein synthesis and is essential for maintaining muscle volume [7,8]. Active vitamin D stimulates VDR expression in satellite cells during muscle regeneration, and VDR and CYP27B1 expression increases significantly following muscle damage. This interaction triggers the intracellular absorption of inorganic phosphates, which are crucial for energy production and muscle contractility [8]. VDR influences various cellular components of skeletal muscle, from embryonic development to post-damage tissue repair and aging modulation. Vitamin D’s effects on musculoskeletal tissue are mediated through two mechanisms: long-term genomic and short-term non-genomic pathways [59]. The genomic pathway involves vitamin D stimulating muscle cell proliferation and differentiation by modulating gene transcription in myoblasts, increasing the synthesis of muscle proteins such as myosin and calcium-binding protein. This pathway involves the binding of vitamin D to VDR, heterodimerization with the retinoic X receptor (RXR), and binding to the vitamin D response element (VDRE) in target genes, regulating inflammatory responses, oxidative stress, cell differentiation, and apoptosis. The non-genomic pathway involves vitamin D binding to nuclear or membrane VDR linked to caveolin-1, leading to the activation of intracellular signaling molecules such as PKC, PI3K, MAPK, calmodulin-dependent protein kinase II, and phospholipase A2 [60]. Both pathways work synergistically to influence muscle contraction and long-term muscle mass and strength (Figure 1). The short-term mechanism regulates calcium-mediated second messenger actions, affecting cytosol-mitochondria interactions to modulate energy metabolism and muscle contraction mechanisms [61]. Vitamin D also prevents the transdifferentiation of myogenic precursors into adipogenic cells, reducing intra- and intermuscular adipose tissue accumulation [62]. An in vitro study on myoblasts exposed to calcitriol demonstrated that it inhibits myostatin secretion [63].

Myostatin is a member of the transforming growth factor-β (TGF-β) superfamily [64]. Myostatin is produced by the skeletal muscle lineage and prevents muscle anabolism and satellite cell synthesis. Being a key negative regulator of muscle mass, it has been associated with reductions in muscle mass. Myostatin effects can be reduced by resistance exercise and myostatin inhibitors, which have been shown to have positive effects on muscle mass [65]. To date have been identified some natural compounds that inhibit myostatin such as curcumin, gingerol, and quercetin [66,67]. However, their mechanism of action has not yet been fully clarified. The addition of 1.25(OH)2 in myoblasts improves VDR expression, reduces cell proliferation, and activates myogenic determination factor 1 (MyoD1), which stimulates myogenic differentiation [63]. In addition, vitamin D regulates the signaling pathways FOXO-3 and Notch, promoting the self-renewal of myoblasts and supporting the satellite stem cell pool [8]. This evidence confirms that vitamin D enhances muscle development and myogenic differentiation.

## 5. Role of Mitochondria of Skeletal Muscle Fibers in Sarcopenia Physiopathology

In skeletal muscles, mitochondria form dynamic, three-dimensional networks to maximize energy efficiency. These energetic organelles are essential in providing various services such as adenosine triphosphate ATP to support muscle contraction through oxidative phosphorylation (OXPHOS), neurotransmitter release and signaling [68], and calcium regulation [69]. Additionally, mitochondria play crucial roles in calcium regulation, amino acid metabolism, and lipid metabolism [70]. The unusual accumulation of calcium ions activates proteases and phosphatases that promote protein degradation, damaging muscle structure and function.

Significant differences in mitochondrial content and functionality exist among different muscle fiber types [71]. Skeletal muscles are classified into fast-twitch and slow-twitch fibers based on contraction speed and further differentiated into slow-oxidative (type I), fast-oxidative (type IIa), and fast-glycolytic (type IIx and IIb) fibers based on metabolic characteristics [72]. Type I muscle fibers exhibit slow contraction speeds and high mitochondrial content, volume, and functionality, making them suited for sustained energy supply. Conversely, type II fibers contract rapidly and are more glycolytic. The proportion of type I to type II fibers in skeletal muscle determines its overall contraction speed [73].

Therefore, diverse muscle fiber types show variances in metabolic demands. Oxidative fibers (I and IIa) display elongated, interconnected mitochondria with a higher fusion rate, while glycolytic fibers (IId/x and IIb) show fragmented, isolated mitochondria with minimal fusion. Since slow-twitch fibers require a slow and sustained energy supply, they possess higher mitochondrial content, higher mitochondrial volume, and greater mitochondrial functionality. The regulation of the mitochondrial network plays a key role in determining muscle size [74]. The safeguard of mitochondrial function and content depends on the mitochondrial quality control system [75], which includes mitochondrial biogenesis, dynamics, mitophagy, and mitochondrial unfolded protein response. Mitochondrial functionality is sustained by fusion proteins such as mitofusin 1 (MFN1) and 2 (MFN2) and optic atrophy 1 (Opa1), along with fission proteins like dynamin-related protein 1 (Drp1), mitochondrial fission factor (Mff), and fission protein 1 (Fis1). Mitochondrial abnormalities caused by the failure of these proteins can result in bioenergetic dysfunctions, oxidative stress, inflammation, and cell death, all of which are features found in a wide spectrum of muscular disorders [76]. In the knockout MFN1 and MFN2 murine model, it was found that the elongated morphology of mitochondria in type IIa fibers needs the normal function of MFN1 and MFN2 for maintenance [77]. In contrast, glycolytic fibers depend on Drp1 expression. Variations in Drp1 expression in glycolytic fibers cause severe defects [78]. Slow-twitch fibers primarily preserve a reticular mitochondrial morphology, retaining high levels of mitochondrial fusion to enhance aerobic metabolic function and meet their energy demands. A disturbed fission process with blockage of Fis1 or Drp1 promotes the formation of elongated mitochondria with increased oxidizing emission [8], and preventing mitochondrial fragmentation originates mitochondrial dysfunction [79]. Over-regulation of fusion or under-regulation of fission could serve to maintain myocyte viability by eliminating compromised mitochondria.

Therefore, the preservation of mitochondrial function depends on the proper balance of mitochondrial dynamic mechanisms, which vary according to muscle fiber type rather than overall muscle type.

Under physiological conditions, a proper amount of free radicals is essential to regulating and ensuring the homeostasis of skeletal myocyte activity and muscle functionality. Ischemic damage alters mitochondrial functions in atrophic perifascicular fibers, characterized by abnormalities of succinate dehydrogenase and myofibers deficient in cytochrome c oxidase (COX) [80]. The amplification of ROS formation due to mitochondrial damage, in turn, induces the expression of type I IFN genes in human myotubes, promoting muscle inflammation [7,8]. On the other hand, ROS production induced by IFN-β in human myotubes promotes mitochondrial damage, creating a vicious circle that worsens muscle failure and disease progression.

### 5.1. Mitochondrial Biogenesis in Sarcopenia

Mitochondrial biogenesis involves various signaling pathways, such as AMPK, p38-MAPK, and Sirt1. The peroxisome proliferator-activated receptor gamma coactivator 1-alpha (PGC-1α) plays a central transcriptional role in mitochondrial biogenesis [81]. Variances in PGC-1α content have been found between fast and slow muscles. For instance, in slow muscles like the soleus, PGC-1α expression is significantly higher than in fast muscles such as the extensor digitorum longus and tibialis anterior [82]. Muscles with a higher content of slow-twitch fibers exhibit higher levels of PGC-1α protein expression, increased mitochondrial content, and enhanced respiratory function compared to muscles with more fast-twitch fibers [83]. PGC-1α induces both NRF1 and NRF2, as well as the expression of mitochondrial transcription factor A (TFAM). TFAM facilitates the transcription and replication of mitochondrial DNA (mtDNA), guaranteeing the correct mitochondrial quantity and function. In sarcopenia, the expression and activity of PGC-1α in muscle cells significantly decline, leading to impaired activation of NRF1, NRF2, and TFAM, decreased replication and transcription of mtDNA, and a reduction in mitochondrial number in muscle cells [84]. These events cause deterioration of muscle strength and function. Experimental research demonstrated that PGC-1α-deficient mice exhibit symptoms of premature aging, such as diminished exercise capacity, metabolic dysregulation, and reduced mitochondrial quantity. In contrast, mice overexpressing PGC-1α show resistance to age-related muscle atrophy [85]. The absence of Nrf2 is associated with the onset of sarcopenia, decreased muscle strength, and a reduction in skeletal muscle mitochondrial quantity. These variations correlate with a reduction in the activity of PGC-1α, NRF1, and TFAM, which are involved in mitochondrial biogenesis and dynamics [86]. Overall, the proper regulation of mitochondrial dynamics and biogenesis is essential for maintaining muscle health and function, particularly in conditions such as sarcopenia.

### 5.2. Mitophagy in Sarcopenia

Mitophagy is an essential mechanism for eliminating damaged mitochondria, thereby ensuring the optimal metabolic function of muscle cells [87]. The process of mitophagy varies among different muscle fiber types. The process of mitophagy varies among different muscle fiber types. The Pink1/Parkin signaling pathway plays a crucial role in mitophagy. Pink1, a serine–threonine protein kinase, is quickly degraded in healthy mitochondria. However, in damaged mitochondria, Pink1 stabilizes and attaches to the outer mitochondrial membrane, recruiting other proteins involved in mitophagy, such as Parkin. Parkin, an E3 ubiquitin ligase, becomes phosphorylated by Pink1 and is subsequently recruited from the cytoplasm to the outer membranes of damaged mitochondria. Mice deficient in Parkin protein exhibit reduced mitochondrial respiration rates, decreased activity of respiratory chain complex subunits encoded by mtDNA, uncoupling phenomena, disordered skeletal muscle mitochondria, reduced levels of Mfn2, which promote mitochondrial fusion, and increased levels of Drp1, which promote mitochondrial fission [88]. Conversely, Parkin overexpression increases mitochondrial quantity and enzyme activity, thereby improving muscle quality and strength [89]. The ubiquitination of proteins such as voltage-dependent anion channel 1 (VDAC1) and the receptor protein of the ubiquitin-binding adapter p62 facilitates the recruitment of the LC3 family to form autophagosomes that mediate mitochondrial autophagy [90]. The LC3 II/I ratio is higher in type I fibers compared to type II fibers.

In cellular stress conditions, the enhancement of free radical levels triggers mitochondrial dysfunction and the activation of muscle autophagy and catabolic pathways [91].

In sarcopenia, mitochondrial stress and ROS release are increased. ROS prompt transition pore opening of mitochondrial membrane permeability (mPTP), reducing mitochondrial levels of β-nicotinamide adenine dinucleotide (NAD) and subsequently causing apoptosis in muscle cells. This results in mitochondrial damage and dysfunction in both muscle and nerve cells [92]. The opening of the mPTP exacerbates cellular stress and alters calcium ion levels necessary for muscle contraction, weakening muscle function. An excessive increase in Ca^2^⁺ within the mitochondria destroys the mitochondrial membrane potential, triggering mitochondrial swelling and pro-apoptotic factor production [93]. Further, mtDNA is susceptible to oxidative stress, causing a domino effect where damage to the electron transport chain further increases oxidative stress [94]. The tumor necrosis factor (TNF-α) strongly stimulates iNOS and affects NO signaling, establishing a mechanistic link between inflammation and mitochondrial impairment. It has been found that TNF-α promotes apoptosis through the death receptor signaling pathway and inhibits mitochondrial biogenesis by downregulating PGC-1α and NRF1 expression in C2C12 myoblasts.

Moreover, oxidative muscles exhibit higher expression levels of the Beclin-1 complex and mitophagy proteins BNIP3 and Parkin compared to glycolytic muscles [95,96]. Additionally, the FoxO3a transcription factor facilitates the formation of mitochondrial autophagosomes in skeletal muscle through BNIP3 mediation [97]. The abundance of FoxO3a is higher in type I muscle fibers, aligning with reports of increased BNIP3 and LC3 II/I levels in oxidative muscles. This suggests that slow-twitch fibers exhibit elevated levels of mitophagy, consistent with their robust oxidative capacity and higher mitochondrial turnover rate. However, excessive or imbalanced mitochondrial autophagy can induce muscle degeneration and developmental abnormalities, underscoring the importance of tightly regulated mitophagy processes [98].

### 5.3. Role of Neuromuscular Junction Mitochondria in Sarcopenia Pathophysiology

Skeletal muscle contraction is initiated by the nervous system, where a signal generated by motor neurons reaches the neuromuscular junction (NMJ). At the NMJ, acetylcholine (ACh) is released and binds to receptors on the sarcolemma of muscle fibers, triggering muscle contraction. The NMJ is the crucial connection point between the motor neuron axon and skeletal muscle fibers, composed of motor nerve endings, peri-synaptic Schwann cells, and muscle fibers [99]. Its primary function is to convert action potentials from presynaptic motor neurons into muscle fiber contractions. Structural and functional alterations at the NMJ are linked to declines in muscular performance. Mitochondria play a critical role at the NMJ, existing on both the pre- and post-synaptic sides [100] and supplying large amounts of adenosine triphosphate (ATP) essential for maintaining NMJ integrity and neuromuscular transmission [101]. Hence, pathological mitochondrial dysfunction exacerbates NMJ deterioration, contributing significantly to sarcopenia [102]. Patients with sarcopenia resulting from mitochondrial myopathy show decreased synaptic vesicles at the presynaptic side, abnormal mitochondria aggregation at both the presynaptic and post-synaptic sides, and reduced NMJs [103]. Animal models with mitochondrial defects confirm that mitochondrial alterations precede NMJ instability and motor neuron degeneration. Aged mice lacking copper-zinc superoxide dismutase show the highest mitochondrial numbers and dysfunction with impaired acetylcholine receptors (AChRs) near the NMJs. In turn, NMJ instability impacts several structural damages, including fragmentation of the post-synaptic site and partial or full degeneration of the innervating α-motor axon nerve, followed by skeletal muscle atrophy and the loss of mobility, culminating with aging [104]. Aging is associated with a progressive reduction in muscle strength and motor performance [105], exacerbated by chronic inflammation, reduced growth factors, and NMJ degeneration [106]. NMJ instability is a significant contributor to sarcopenia pathophysiology [107]. In primary sarcopenia, aging disrupts NMJ structure and function, leading to fragmentation and degeneration and contributing to muscle weakness [108]. Secondary sarcopenia, observed in conditions such as amyotrophic lateral sclerosis (ALS) [109] and myasthenia gravis, also exhibits NMJ abnormalities [110]. It has been proposed that mitochondrial dysfunction might precede pathological changes in muscles and NMJ instability in neuromuscular diseases [111]. An experimental study in a murine model with mitochondrial myopathy due to unstable mtDNA confirmed that mitochondrial alterations in skeletal muscles anticipate NMJ instability and motor neuron degeneration [112]. It was also found that transgenic mice overexpressing fused in sarcoma (FUS), a marker associated with FUS-related ALS and frontotemporal dementia, exhibited mitochondrial anomalies and disruptions in the NMJ [113]. Moreover, both single and multiple deletions of mtDNA can lead to alterations in myelin sheaths and crystal-like inclusions in Schwann cells at NMJs [114]. In addition, mitochondrial free radicals are essential effectors in NMJ alterations linked to sarcopenia [115]. If the over-production of reactive species due to mitochondrial dysfunction is not neutralized by endogenous or exogenous antioxidants, it can promote oxidative damage to cellular infrastructure and function [116]. Mice with a muscle-specific SOD1 mutation develop fiber atrophy, loss of strength, and mitochondrial dysfunction [117], thus becoming a potential model for sarcopenia. This SOD1 mutation in skeletal muscle induces loss of mitochondrial inner membrane potential (MIM) near the NMJ, disrupts NMJ structure, impairs transmembrane potential in pre- and post-synaptic mitochondria, and hampers propagating Ca^2+^ waves and signaling in skeletal muscle [118]. PKCϴ is a redox-sensitive kinase expressed in the post-synaptic region of the NMJ. It plays a crucial role in eliminating AChR in skeletal muscle and mediating nerve-muscle interactions [119]. Investigating the role of PKC_ϴ_, it was shown that it may activate downstream pathways related to NMJ degradation [111]. Furthermore, inhibiting PKCϴ can preserve redox signaling in muscle neurons and the NMJ, potentially preventing sarcopenia. Additionally, evidence for retrograde signaling from skeletal muscle to the NMJ is provided by studies showing that transgenic expression of PGC-1α leads to morphological and functional remodeling, including improved NMJ structure [120]. Aging rats show intense structural changes in the mitochondria of distal motor axon terminals, but not of motor neurons within the ventral horn of the spinal cord [121]. The local specificity of mitochondrial structural changes is crucial for the expression of apoptotic markers and their co-localization with retrograde transport proteins in the soma, which is representative of an initial degenerative phase starting distally at the NMJ. Structural changes in the NMJs can disrupt functional integrity and mitochondrial health, further exacerbating sarcopenia [122]. Furthermore, nuclear-encoded mitochondrial messenger RNAs have been found in the axon at the NMJs, where they are transferred close to the mitochondria within the axon [123]. The mechanism of autophagy, essential for removing unnecessary or dysfunctional components, decreases with aging. Recent research shows that inhibiting autophagic flow increases oxidative stress in young muscles, impairs muscle strength generation, and induces mitochondrial dysfunction and NMJ instability [124]. It has been reported that scavenging free radicals can efficiently avoid mitochondrial dysfunction and consequent NMJ destruction associated with muscle atrophy due to denervation. Optional nutritional intake is an efficient approach to preventing the negative effects of oxidative stress on NMJ. Antioxidative bioactive compounds such as resveratrol and vitamin D are proving competent in NMJ prevention or alleviation and mitochondrial damage in sarcopenia, as they exert beneficial effects also through their influence on NMJ.

## 6. Resveratrol Molecular Mechanisms in Mitochondria Health

**Figure 2:**
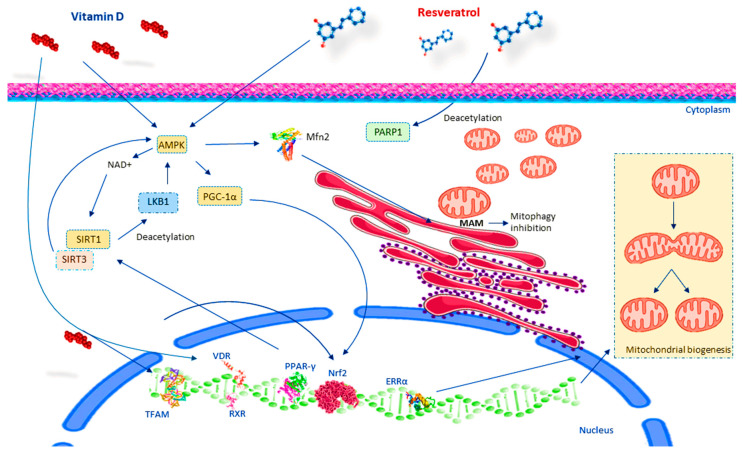
Resveratrol and vitamin D action in mitochondria health. Abbreviations: adenosine monophosphate protein kinase (AMPK); estrogen-related receptor-α (ERR-α); liver kinase B1 (LKB1); mitochondria-associated endoplasmic reticulum membrane (MAM); nuclear respiration factors 1 and 2 (NRF-1, NRF-2); peroxisome proliferator-activated receptor gamma (PPAR-γ); retinoid X receptor (RXR); sirtuin 1 (SIRT-1); sirtuin 3 (SIRT-3); transcription factor A mitochondrial (TFAM); vitamin D receptor (VDR).

Several pieces of evidence report that resveratrol can prevent muscle wasting, enhance muscle protein synthesis, decrease muscle protein degeneration, and mitigate the atrophy of skeletal muscle fibers [33,125]. These health benefits are primarily attributed to resveratrol’s ability to increase energy expenditure by stimulating mitochondrial respiration, which is crucial for combating sarcopenia, a condition characterized by defective mitochondrial function. In humans, resveratrol combined with exercise has been shown to reduce sarcopenia more effectively than exercise alone. This combination enhances maximal oxygen consumption and significantly improves mitochondrial volume density [126]. In addition, resveratrol modulates genes linked to mitochondrial morphology and influences genes associated with mitochondrial-regulated apoptotic signaling, which is essential for eliminating dysfunctional organelles [32]. Experimental evidence has demonstrated that resveratrol can increase mitochondrial biogenesis by acting on its main effectors, such as PGC-1α, SIRT1, AMPK, estrogen-related receptor-α (ERR-α), telomerase reverse transcriptase (TERT), TFAM, NRF-1, and NRF-2, through multiple mechanisms of action [127]. Acting as an SIRT1 activator, resveratrol action is dependent on SIRT1. Sirtuin deacetylases control protein acetylation/deacetylation within the mitochondria, influencing mitochondrial function [128]. It has been observed that deletion of SIRT1 in adult mice impairs resveratrol’s ability to increase biogenesis and mitochondrial function [129,130]. Reduced SIRT1 activity in aging is linked to reduced oxidative capacity and ATP synthesis. Once resveratrol activates SIRT1, it triggers the deacetylation of several metabolic transcriptional regulators in vivo, including PGC-1α, which is a target of SIRT1 [131]. PGC-1α is one of the main coordinators of mitochondrial biogenesis [132]. Following deacetylation by SIRT1, PGC-1α increases the activating capacity of numerous transcription factors and nuclear receptors controlling mitochondrial gene expression [133]. Therefore, resveratrol induces increased mitochondrial content in important metabolic tissues, such as skeletal muscle or brown adipose tissue. In cultured myocytes, the effect of resveratrol on PGC-1α activity is limited if SIRT1 is inhibited [134]. Treatment with resveratrol leads to PARP-1 deacetylation [135]. It has been proposed that AMPK is the initial trigger in the SIRT1-PARP loop [136], with AMPK activating SIRT1 through increased NAD+ levels and SIRT1 activating AMPK by deacetylating LKB1 [137]. AMPK inhibition interrupts SIRT1-mediated PGC-1α deacetylation, affecting respiratory activity [138]. It has been found that resveratrol stimulates AMPK in SIRT1-deficient cells, whereas reduced AMPK activity suppresses resveratrol-induced SIRT1 activation [139]. Moreover, resveratrol acts as an inhibitor of mitophagy through AMPK-Mfn2 signaling pathway, specifically regulated by mitochondria-associated endoplasmic reticulum membranes (MAMs) [140]. MAMs consist of various protein complexes, such as VAPB-PTIP51, IP3R-GRP75-VDAC, BAP31-Fis1, and Mfn1/2, the mitochondrial fusion protein that governs mitochondrial morphology [141]. It is involved in calcium homeostasis, lipid metabolism, protein translocation between mitochondria and the ER, the formation of inflammatory vesicles, autophagy, ER stress, and mitochondrial morphology [142], and so MAMs are counted among the main regulators of autophagy [141]. It has been reported that resveratrol inhibits mitophagy by regulating MAMs through AMPK-Mfn2. Studies show that resveratrol can improve mitochondrial elongation and affect mitophagy via the classical PINK1/Parkin-mediated mitophagy pathway [143]. Another study showed that resveratrol inhibits mitophagy via the PINK1-Parkin and AMPK-Mfn2 pathways under zinc-deficient conditions [140]. TPEN significantly increased LC3 expression and lysosomal and mitochondrial co-localization. Resveratrol treatment significantly inhibits TPEN-induced effects and so can regulate TPEN-induced mitophagy through MAMs (Figure 2).

### The Effect of Resveratrol on NMJ Function

Some evidence suggests that resveratrol can target the presynaptic component of the NMJ and accelerate the restoration of damaged and disease-affected synapses. It has been shown that resveratrol controls neuromuscular communication, improving motor coordination and traction force in mice fed a high-fat diet. In middle-aged rats, resveratrol treatment induces the formation of new synapses [144]. A resveratrol-rich diet in mice delays age-dependent NMJ structural modifications by reducing its fragmentation and denervation [145]. Resveratrol also increased the number of post-synaptic sites, “rejuvenating” the architecture formed in C2C12-derived myotubes. This observation supports the idea that resveratrol preserves motor function by protecting NMJs and muscle fibers. Moreover, resveratrol may affect other cells that are involved in the health of muscle fibers and NMJs, including motor neurons, Schwann cells, and satellite cells [145]. Furthermore, resveratrol exerts neuroprotective effects by modulating the expression/activity of specific neuroprotective proteins during acute physiological stress [146]. It is also able to increase neurotrophic factor production, such as nerve growth factor (NGF) and glial cell line-derived neurotrophic factor (GDNF) production [147,148]. Resveratrol induces signaling components to maintain the structure of the NMJ as well as muscle fibers. Resveratrol can inhibit PKC [149] and also induce the expression of genes involved in mitochondrial biogenesis and oxidative phosphorylation through PGC-1-α [138]. As a result, muscle fibers from treated animals have an improved oxidative level with increased oxidative type I fibers, which are resistant to fatigue [150]. Resveratrol in skeletal muscles activates sirtuin and the mechanistic target of rapamycin (mTOR) [151] pathways, which affect the aging of NMJ located at the pre- and post-synaptic regions, indicating that it uses molecular mechanisms that act both overall in muscle fibers and precisely on the post-synaptic region of the NMJ. Moreover, resveratrol intervenes on the AMPK-SIRT-1 pathway to activate several signaling pathways, inducing myofiber remodeling. RES supplementation mitigates age-dependent fiber area reduction [35]. Interestingly, the administration of resveratrol in rats undergoing hind limb suspension improved the cross-sectional area of type II fibers in response to refilling, most likely by decreasing pro-apoptotic signals [37] (Figure 2).

## 7. Vitamin D Molecular Mechanisms in Mitochondria Health of Skeletal Muscle

**Figure 3:**
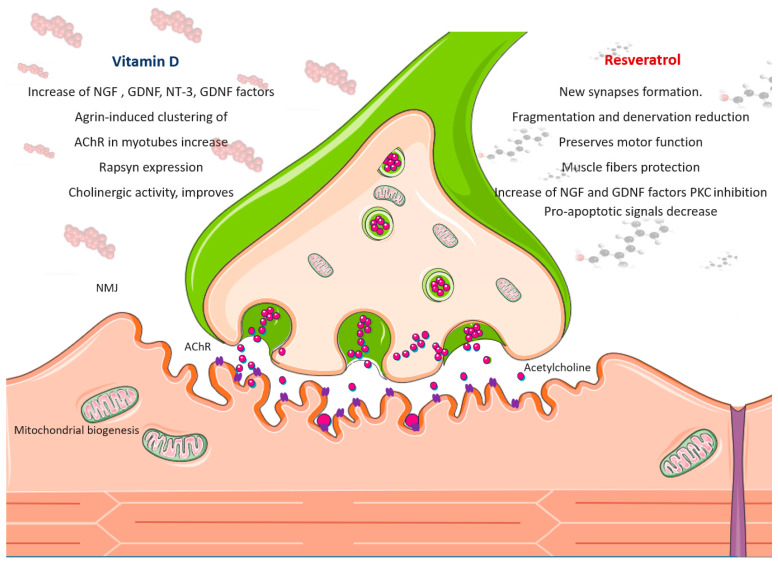
Schematic presentation of resveratrol and vitamin D effect on neuromuscular junction (NMJ). Resveratrol and vitamin D induce myofibers remodeling and mitochondrial myogenesis and act on various molecular pathways that influence the NMJ at the pre- and post-synaptic levels. Resveratrol and vitamin D improve cholinergic activity. At the presynaptic terminal of the motor nerve, where vesicles fuse with the terminal membrane and release the neurotransmitter acetylcholine (in pink), the post-synaptic skeletal muscle membrane exhibits acetylcholine receptors (AChRs, in violet) concentrated on the crests.

The role of vitamin D in skeletal muscle extends beyond its traditional functions in bone health, encompassing crucial aspects of mitochondrial dynamics, energy metabolism, and oxidative stress regulation [152]. It is well known that vitamin D regulates Ca_2_^+^ homeostasis in both skeletal muscle and mitochondria. Ca_2_^+^ is fundamental for muscle energy metabolism as it interacts between mitochondria and the cytosol [153]. In mitochondrial dysfunction, intracellular Ca_2_^+^ increases significantly [154]. Vitamin D deficiency (VDD) modifies the kinetics of muscle contraction by reducing Ca2+ reuptake in the sarcoplasmic reticulum, resulting in the maintenance of the relaxation phase of muscle contraction. Insufficient levels of mitochondrial Ca_2_^+^ cause disorders of cellular metabolic homeostasis [155]. In addition, VDD increases ROS-mediated cytotoxicity and is linked to mitochondrial respiratory failure. Therefore, VDD can promote the exacerbation of muscle damage and atrophy due to the excessive production of mitochondrial ROS [155], oxidative impairment, and reduction of ATP [155]. In skeletal muscle, VDD has an impact on nitrosative stress, protein and lipid peroxidation, and the decline of antioxidant enzyme activity [8]. In the C2C12 1 cell line, vitamin D treatment induces a reduction in ROS synthesis, protein ubiquitination, lipid and protein oxidation, intracellular impairment, muscle proteolysis, and atrophy. In paraspinal muscle, vitamin D increases the activity of GPx, SOD, and mitochondrial biogenesis markers [156]. In skeletal muscle cells, vitamin D treatment promotes oxygen consumption rate (OCR) and ATP generation [157]. In VDD subjects, vitamin D supplementation increases the rate of mitochondrial oxidative phosphorylation [158]. However, these data are conflicting. In other studies, it was observed that vitamin D does not induce an increase in OCR in the mitochondria. Therefore, it has been hypothesized that the effects of OCR may be dependent on VDR [158]. The explanation of the mechanism by which vitamin D regulates oxidative stress may depend on its effect on the regulation of mitochondrial activity and dynamism. Nrf-2 is an essential transcription factor that intervenes in antioxidant defense pathways [159]. The under-regulation of Nrf-2 generates the collapse of the antioxidant defense system [160]. Vitamin D supplementation activates VDR [161] and stimulates the antioxidant pathway Nrf2–Keap1 [159]. Further vitamin D supplementation enhances skeletal muscle health by upregulating VDR, SIRT1, and SIRT3 [162]. SIRT1 and SIRT3 are NAD+-dependent deacetylases involved in mitochondrial function, oxidative stress response, and metabolism [163]. VDR and SIRT1 interact directly. VDR binds to the SIRT1 promoter or other proteins in the transcription complex. Moreover, VDR and SIRT1 regulate each other via epigenetic modifications [164]. VDR, SIRT1, and SIRT3 upregulate vitamin D-induced AMPK and AKT phosphorylation [165]. SIRT3 is a mitochondrial sirtuin that regulates AMPK by deacetylating LKB1 and other AMPK-regulating proteins, leading to AMPK phosphorylation and activation [166]. AMPK supports cellular energy metabolism and influences mitochondrial biogenesis [167]. Increased AMPK improves muscle protein production and, together with SIRT1, acts to enhance muscle growth [168]. AMPK/AKT phosphorylation indicates the existence of the VDR/SIRT1/SIRT3 axis in regulatory mechanisms in numerous cellular processes linked to skeletal muscle development. Activating VDR/SIRT1/SIRT3 vitamin D induces the expression of OXPHOS-related mRNA and protects mtDNA content. Moreover, VDR operates as an upstream regulator of the expression and activity of sirtuins. VDD impacts muscle health by decreasing mtDNA content and the expression of critical biogenesis-related factors such as PGC-1α, NRF1, NRF2, PPARα, and COXIV.SIRT3 activation, facilitated by PGC-1α under energy stress, enhances mitochondrial function and OXPHOS [163]. SIRT3 deficiency causes acetylation at over 400 mitochondrial sites that are involved in all aspects of mitochondrial biological functions, including OXPHOS [169]. This leads to mitochondrial hyperacetylation and reduced PGC-1α targets (NRF-1 and mtTFA), whereas SIRT3 overexpression increases mtDNA content [169]. SIRT3 deacetylates and activates subunits of the OXPHOS complex (complexes I, II, III, IV, and V) in the electron transport chain and significantly influences mitochondrial biogenesis, gene expression, and activation of oxidative phosphorylation components [169]. In vitro and in vivo studies indicate that 1,25VD3 activation of VDR, SIRT1, and AMPK reduces oxidative stress, and mitochondrial dysfunction [170]. Being the main regulators of the oxidative capacity of muscle fiber and mitochondrial biogenesis, both AMPK and Sirt1 induce transcription of PGC-1α, which in turn helps various transcription factors regulate the mitochondrial content of the tissue. It is well known that the AMPK-SIRT1-PGC-1α signaling pathway acts as an energy-sensing network that is crucial for mitochondrial biosynthesis, energy metabolism, and oxidative stress. AMPK is a core component of the AMPK-SIRT1-PGC-1α signaling pathway that regulates the switch between anabolic and catabolic metabolism. Vitamin D upregulates VDR, SIRT1, and SIRT3 and restores AMPK and AKT activation, leading to improved muscle development, myotube diameter, and MyHC expression by reducing FoxO3a transcriptional activity. The transcriptional activity of FoxO3a is regulated by post-translational modifications such as phosphorylation and deacetylation, which are responsible for its activation and deactivation [171]. VDR promotes FoxO3a deacetylation through SIRT1 and SIRT3 expression, whereas AMPK activation induces FoxO3a phosphorylation [172]. FoxO3 phosphorylation, reducing its transcriptional activity, prevents muscle protein degradation through the FoxO3a/MAFbx/MuRF1 pathway. Vitamin D preserves skeletal muscle health by regulating the activity of FoxO3a and its downstream targets. Vitamin D deficiency in rats results in elevated protein breakdown and MAFbx/MuRF1 expression, which can be reversed by restoring vitamin D levels [61]. Reestablishing optimal vitamin D levels alleviates myopathy symptoms and improves muscle mitochondrial oxidative capability [173] (Figure 3).

### The Effect of Vitamin D on NMJ Function

While direct evidence for vitamin D’s role in NMJ homeostasis remains limited, preclinical and clinical studies suggest it benefits the neuromuscular system. Vitamin D appears to influence the expression of several neurotrophic factors crucial for nerve cell growth and survival [174]. These include NGF, neurotrophin-3 (NT-3), GDNF, and the C-terminal fragment of agrin [175,176,177,178]. Notably, vitamin D deficiency in the nervous system is linked to a decrease in neurotrophic factor production [179].

Studies using animal models further support this connection. Altered agrin expression in mice led to NMJ fragmentation, similar to what’s observed in aged NMJs and premature sarcopenia. Interestingly, vitamin D treatment in these models increased the agrin-induced clustering of AChR in myotubes [180]. In addition, vitamin D signaling through the VDR also regulates Rapsyn expression, another protein involved in AChR clustering [181]. Previously, it has been reported that vitamin D deficiency or both vitamin D and Ca^2+^ deficiency may cause alterations in the structure and function of the NMJ and the absence of a persistent stress response in muscles [182]. In rat models, vitamin D treatment improves cholinergic activity, promotes NGF production, and counteracts neural aging [183]. In rodents, vitamin D deficiency, causing modifications in the genomic and proteomic profile of muscle, particularly in NMJ-related genes and proteins [183], affects muscle performance [183]. Vitamin D supplementation promotes neuromuscular remodeling and repair in both cell and animal models, aiding recovery from injury and addressing the effects of aging [184]. Therefore, vitamin D-regulating NMJ biomarkers exert beneficial effects on muscle function (Figure 2).

## 8. Concluding Remarks and Future Direction

Several factors contribute to muscle decline and the onset of sarcopenia, including structural and physiological decreases in muscle tissue, increased oxidative stress, inflammation, and mitochondrial dysfunction. Experimental evidence reviewed here indicates that resveratrol and vitamin D share similar molecular mechanisms (Figure 2). Both compounds promote healthy neuromuscular aging by exerting myotrophic, neurotrophic, and anti-inflammatory effects. Both promote inhibition of the NF-κB pathway and COX-2 pathways, TLR expression, and NLR family pyrin domain-containing 3 (NLRP3)-inflammasome activation and facilitate anti-inflammatory M2 polarization and Treg activation [31]. Furthermore, the regulation of mitochondrial health by resveratrol and vitamin D may impact satellite cell activity and self-renewal, potentially enhancing muscle regeneration. These findings suggest that using resveratrol and vitamin D together could have a synergistic beneficial effect. In particular, both resveratrol and vitamin D control oxidative stress and regulate mitochondrial biogenesis and NMJ function, often influencing the same signaling pathways. Therefore, further insights on their synergistic contribution in maintaining and restoring muscle strength could demonstrate their effectiveness in focusing their influence on mitochondrial health and evaluating their potential clinical application to slow the progression of sarcopenia.
